# Patients' prognosis with congenital heart disease followed by ten years: survival and associated factors

**DOI:** 10.1590/1984-0462/2024/42/2023134

**Published:** 2024-02-12

**Authors:** Daniélle Bernardi Silveira, Rodrigo da Silva Batisti, Liana Vitória Marchezi, Beatriz Felipe da Rocha, Ernani Bohrer da Rosa, Jamile Dutra Correia, Leonardo Leiria de Moura da Silva, Rafael Fabiano Machado Rosa, Paulo Ricardo Gazzola Zen

**Affiliations:** a Universidade Federal de Ciências da Saúde de Porto Alegre, Irmandade da Santa Casa de Misericórdia de Porto Alegre, Porto Alegre, RS, Brazil.

**Keywords:** Congenital heart defects, Infant mortality, Mortality, Infant, newborn, Risk factors, Cardiopatias congênitas, Mortalidade infantil, Mortalidade, Recém-nascido, Fatores de risco

## Abstract

**Objective:**

To evaluate the prognosis and influence of associated factors in patients with congenital heart disease admitted for the first time to the Intensive Care Unit of the Hospital da Criança Santo Antônio/Irmandade da Santa Casa de Misericórdia de Porto Alegre, especially those factors associated with death.

**Methods:**

Patients were prospectively and consecutively allocated over a period of one year (August 2005 to July 2006). Now, 15 years after the initial selection, we collected data from these patients in the database of the Cytogenetics Laboratory of the Universidade Federal de Ciências da Saúde de Porto Alegre and in the medical records of the hospital.

**Results:**

Of the 96 patients, 11 died and 85 were alive until 20 years old. Four patients died in the Intensive Care Unit. The survival probability up to 365 days of life was 95.8%. The survival assessment identified that the deaths occurred mainly before the patients completed one thousand days of life. We found that complex heart disease was independently associated with an odds ratio of 5.19 (95% confidence interval — CI:1.09–24.71; p=0.038) for death.

**Conclusions:**

Knowledge about the factors that interfere with the prognosis can be crucial in care practice planning, especially considering that congenital heart disease is an important cause of mortality in the first year of life.

## RESUMO

**Objetivo:**

Avaliar o prognóstico e a influência de fatores associados em pacientes com cardiopatia congênita internados pela primeira vez na Unidade de Terapia Intensiva do Hospital da Criança Santo Antônio/Irmandade da Santa Casa de Misericórdia de Porto Alegre, principalmente aqueles fatores associados ao óbito.

**Métodos:**

Os pacientes foram alocados prospectiva e consecutivamente por um período de um ano (agosto de 2005 a julho de 2006). Agora, 15 anos após a seleção inicial, coletamos dados desses pacientes no banco de dados do Laboratório de Citogenética da Universidade Federal de Ciências da Saúde de Porto Alegre e nos prontuários do hospital.

**Resultados:**

Dos 96 pacientes, 11 faleceram e 85 permaneceram vivos até completar 20 anos. Quatro pacientes morreram na Unidade de Terapia Intensiva. A probabilidade de sobrevida até 365 dias de vida foi de 95,8%. A avaliação da sobrevida identificou que os óbitos ocorreram principalmente antes de os pacientes completarem mil dias de vida. Verificamos que a doença cardíaca complexa foi independentemente associada a um *odds ratio* de 5,19 (intervalo de confiança — IC95% 1,09–24,71; p=0,038) para morte.

**Conclusões:**

O conhecimento dos fatores que interferem no prognóstico pode ser fundamental no planejamento da prática assistencial, principalmente considerando-se que as cardiopatias congênitas são importante causa de mortalidade no primeiro ano de vida.

## INTRODUCTION

Congenital heart diseases (CHD) are defined as any abnormality in the function or structure of the heart that appears in the first eight weeks of gestation. Such anatomophysiological variations occur due to alterations in the embryonic development of the structure of the heart. CHD is the most common congenital problem and a leading cause of death among malformations.^1,2^

CHD may be broadly grouped into two major categories: morphological anomalies including developmental defects resulting in structural malformations and functional irregularities, heart rhythm disturbances and cardiomyopathies.^1^ They can also be divided into two groups: acyanotic and cyanotic heart defects. The acyanotic group is characterized by an injury that does not have the capacity to produce cyanosis, since there is no obstruction of venous blood in the systemic circulation. In turn, the cyanotic group presents a lesion capable of producing cyanosis, as deoxygenated blood enters the systemic circulation. The risk of fetal mortality in cyanotic CHD is higher and correction, even when partial, significantly decreases the fetal and neonatal risk.^2^

About 20 to 30% of patients with CHD who do not receive adequate treatment die in the first month of life due to heart failure and/or hypoxic crises, and about 50% die by the end of the first year of life.^3–5^

Knowledge about the prognosis of CHD supports the obtaining of indicators related to good care practices and specific outcomes, such as death. This can provide better public policies aimed at this group of children, in addition to the best health services practices.^6^ Therefore, the determination of aspects related to the outcome, including death, can have important repercussions on the management and treatment of patients, as well as on their living conditions. In the present study, we investigated the prognosis and the influence of associated factors in patients with congenital heart disease in a sample of patients from Southern Brazil.

## METHOD

The sample consisted of 96 patients with CHD who were hospitalized for the first time in the Intensive Care Unit (ICU) of the Hospital Santo Antônio (HCSA)/Irmandade da Santa Casa de Misericórdia de Porto Alegre (ISCMPA). We proposed the follow-up of patients described by Rosa et al.,^7^ whose patients were prospectively and consecutively allocated over a period of one year (August 2005 to July 2006). The present study is retrospective, applying a clinical protocol and collecting patient data from the database of the Clinical Genetics Service of the Universidade Federal de Ciências da Saúde de Porto Alegre (UFCSPA) and from the medical records of the HCSA. The study was approved by the ethics committee of the institution and those responsible agreed to participate. All institutions are located in Porto Alegre, Southern Brazil.

The protocol contains identification and demographic data, parents' education, gestational history and birth data, survival or death, hospitalizations, professionals involved in hospital care procedures performed, use of invasive or non-invasive support procedures, tests performed, neuropsychomotor development and karyotype.

The Statistical Package for the Social Sciences (SPSS) for Windows (version 28.0) software and WinPEPI Programs for Epidemiologists for Windows (version 11.65) were used for assembling electronic spreadsheets and data analysis. Numerical variables were described by mean and standard deviation or median and interquartile range. Categorical variables were described by absolute and relative frequencies. Analyses were also carried out using chi-square test, Fisher's exact test and Student's t test, when appropriate, as shown in the results section. Mann-Whitney for median comparison and Kaplan-Meier method were used to estimate the survival curve. To control for confounding factors, a logistic regression model with backward extraction was used. The criterion for entering a variable in the multivariate model was that it had a value of p<0.20 in the bivariate analysis and variables with p<0.10 were maintained in the final model. The level of significance was set at 0.05.

## RESULTS

The general description of the variables of the 96 patients is in Table 1. Of these, male gender was 51 (53.1%), mean (SD) weight at birth (n=72) of 3104.03 (627.8) grams; and mean (SD) length at birth (n=63) of 47.8 (2.8) cm. Low birth weight was found in 12.5%.

**Table 1:** Description of cardiac alterations verified in our sample.

CHD	n	%
Complex	31	32
Cyanotic	30	31
Septal defects	36	37.5
Outflow tract defects	21	22
Ventricular septal defects	18	19
Tetralogy of fallot	11	11.5
Left obstructive defects	11	11.5
Atrioventricular septal defects	10	10
Right obstructive defects	6	6
Dextro-transposition of the great arteries	6	6
Heterotaxia	2	2

CHD: congenital heart diseases.

As for gestational age, most patients were born at term between 37 and 42 weeks (60–83.3%), followed by preterm <37 weeks (10–13.9%) and post-term >42 weeks (2–2.8%). Of these, 49.3 % were born by normal delivery and cesarean section and 90.6% performed prenatal care. Threatened miscarriage and maternal disease were reported by seven and 50 mothers, respectively.

As for the cytogenetic investigation, 77 patients underwent karyotyping due to suspected genetic syndrome. Of these, 15 presented numerical and one presented structural change, the main diagnosis being of Down syndrome (14 patients).

The characteristics of hospital care received by patients are described in Table 2. The median age at first hospitalization was 421 days (193–1330). The number of hospitalizations, days of hospitalization, attendance in the Intensive Care Unit (ICU) or during hospitalization, professionals involved in the consultations and imaging and laboratory tests are detailed in Table 2.

**Table 2:** General characteristics of the sample.

Variables	Median (P25–P75)
Age at first hospitalization (days)	421 (193–1330)
Hospitalizations	2 (1–3)
Days of hospitalization	13 (8–24)
Attendances during hospitalization	235 (153–380)
Attendances in the ICU	148 (100–282)
Clinical inpatient services	70 (40–98)
Number of professionals who provided care at the first hospitalization*	4.2 (1.0)
Professionals who provided care in the ICU*	3.95 (1.2)
Professionals who provided care in clinical hospitalization	3.6 (1.0)
Nurses in the ICU	45 (24–105)
Nurses in clinical hospitalization	6 (3–10)
Nursing technicians in the ICU	70 (47–139)
Nursing technicians in clinical hospitalization	47 (26–73)
Doctors in the ICU	24 (16–48)
Doctors in clinic hospitalization	9 (5–17)
Dressings in hospitalization	17 (8–30)
Imaging tests during hospitalization	6 (4–11)
Laboratory tests during hospitalization	64 (52–92)
Death (days)	6237 (5827–7060)

ICU: Intensive Care Unit.

* Mean±standard deviation.

Most of the patients came from the interior of the state 39 (54.9%), 23 (32.4%) from the capital Porto Alegre and the metropolitan area, and nine (12.7%) from other states. The median age at first ICU admission was 421 days (interquartile range of 193–1330); the median for death in days was 6236.5 days (interquartile range of 5826.75–7059.75).

Of the 96 (100%) patients, 11 (11.5%) died and 85 (88.5%) were alive until completing 7300 days of life, that is, for more than 20 years. Of the patients who died, four (36.36%) died in the ICU, one (9.09%) in clinical hospitalization and six (54.54%) died in an unknown location.

**Figure 1:**
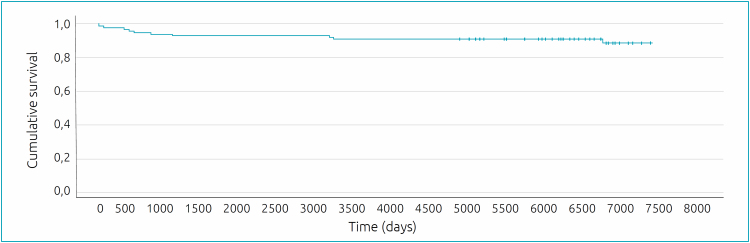
Evaluation of survival with the Kaplan-Meier method.

Survival assessment (Figure 1) using the Kaplan-Meier method identified that deaths occurred mainly before the patients reached one thousand days of life. Of the 11 deaths, eight occurred before the patients reached one thousand days of life and three occurred after 3060, 3102 and 6661 days of life. The probability of survival up to 365 days of life was 95.8%, up to a thousand days of life it was 91.7%, up to 3102 days of life it was 89.6%, maintaining this percentage up to 6660 days of life, and up to the follow-up period of 7300 days of life it was 86.9%.

In logistic regression, complex heart disease was independently associated with an odds ratio (OR) of 5.19 (95% confidence interval — CI 1.09 to 24.71; p=0.038) for death. Although the number of medical appointments during clinical hospitalization was not statistically significant, there was a tendency for a 3.9% increase in the chance of death for an increase of one medical appointment during clinical hospitalization (OR=1.03; 95%CI 0.99 to 1.08; p=0.091).

## DISCUSSION

In our study, the most frequent cardiac alterations were complex, cyanotic and septal defects (Table 1). Low weight, cyanotic CHC and complex CHD presented a "p" significantly associated with death. Furthermore, deaths occurred mainly before the patients reached a thousand days of life and the probability of survival up to 365 days of life was 95.8. Complex heart disease was independently associated with death and the number of medical appointments during clinical hospitalization had a tendency to increase the chance of death.

Our sample consisted of 96 patients with CHD, similar to other studies.^8–13^ Studies that described much larger samples were performed using epidemiological databases.^6,14–16^ The present work demonstrated the predominance of CHD in males (53.1%), in agreement with others publications in Brazil.^6,8,11,13,15–17^ Regarding consanguinity, we found only one case (1.5%). This is similar to the study by Pinto et al.,^18^ where consanguinity was also present in one case (3%); and different from Araújo et al.,^15^ who identified consanguinity in 70.7% of cases. In our study, patients who died did not have consanguineous parents.

In our study, more than 90% of the cases were not diagnosed with CHD during prenatal care, similar to Correia et al.^9^ and different from Already, Wolter et al.^10^ (46.8%). Furthermore, in our study, 10.8% of the cases had threatened miscarriage during pregnancy (Table 1). One patient who had a threatened miscarriage during pregnancy died. Analysis of this variable in other studies was not found.

Maternal diseases during pregnancy are identified as risk factors for CHD and may be associated with the use of over-the-counter medications, increasing the chance of CHD.^19^ In our study, we also found 52.1% of maternal illnesses during pregnancy. Four patients died whose mothers had the disease during pregnancy, but the analysis did not show a significant difference.

Premature newborns, with low birth weight (<2500 grams) and presence of comorbidities have a higher risk of mortality related to CHD.^12^ In our study, patients weighing <2500 grams at birth died more (p=0.026). Furthermore, 36.4% of patients with CHD, prematurity and low birth weight died, which is similar to other studies.^15,20^

In our study, the presence of Down Syndrome associated with CHD was found in 14 cases (14.9%), in line with other studies.^13,17^ Amorim et al. found that isolated heart disease occurred in 37.2% of cases among live newborns and 18.7% among stillborns; and it was associated with syndromes in 23.1% of live newborns and 32% of stillborns.^14^

We did not find statistically significant differences or studies that explore the information contained in Table 3, such as number of consultations, number of professionals who performed consultations, professionals, blood transfusions, dressings performed and number of laboratory and imaging tests performed during hospitalizations. The description of these variables in other studies may help to outline the structure of care, as well as indicate measures that may help to reduce the length of hospital stay and deaths.

**Table 3:** Characteristics of patients who died.

Variables		Death n (%)		p
		Yes	No	
Female		8 (73)	43 (51)	0.288
Had prenatal care		10 (91)	77 (91)	1.000
No consanguinity		9 (100)	56 (98.2)	1.000
Threatened miscarriage		1 (11)	6 (11)	1.000
Maternal disease		4 (36)	46 (54)	0.430
Prenatal diagnosis		2 (22)	7 (78)	0.186
Vaginal birth		6 (55)	31 (48)	0.962
Gestational age				
	<37 weeks	4 (36)	6 (10)	0.058
	37 to 42 weeks	7 (64)	53 (87)	
	>42 weeks	0 (0)	2 (3)	
Low weight <2500 grams at birth		4 (36)	5 (8)	**0.026**
Down's Syndrome		1 (10)	13 (16)	1.000
Presence of cyanotic CHD		7 (64)	23 (27)	**0.032**
Presence of complex CHD		8 (73)	23 (27)	**0.004**
Numerical change in karyotype		1 (10)	14 (17)	1.000
Structural change in karyotype		0 (0)	1 (1)	1.000
Complications during hospitalization		7 (78)	37 (45)	0.081
Blood transfusion in hospitalization		7 (70)	33 (39)	0.091
Birth weight (grams)*		2891 (788)	3142 (594)	0.226
Length at birth (cm)*		46.3 (2.7)	48.0 (2.7)	0.082

CHD: congenital heart disease.

* Mean ± standard deviation.

Bold indicates statistically significant p-values.

CHD patients who died had a mean number of hospitalization days 47 (15–101) longer than patients who did not die 12 (8–19) (p=0.010). In addition, patients who died remained hospitalized for more days (Table 3). It appears that there is a greater demand for care on the part of multidisciplinary teams, when CHD are complex. Of the patients who died, 77.8% had complications during hospitalization, with no statistically significant difference (p=0.081).

Acyanotic CHD are more frequent than cyanotic ones. This piece of data is confirmed in this study, with 68.8% of CHD cases being classified as acyanotic.^2,11,17^ Our study demonstrated a significant association (p=0.032) between cases of cyanotic CHD and death. In addition, complex CHD also showed statistical difference (p=0.004) related to death (Table 3). The risk of fetal mortality in cyanotic CHD is higher, but its correction, even when partial, significantly decreases the fetal and neonatal risk.^2^

In the first hospitalization of patients who died the median age was 193 days, with a statistically significant difference from the median of patients who did not die (p=0.023). For Catarino et al., the median age at the first hospitalization was 23 days.^6^ It is believed that the later hospitalization in our study may have contributed to mortality.

In our study, deaths occurred mainly before the patients reached a thousand days of life. Nina et al. described that 20 to 30% of children with untreated CHD die during the first month of life.^3^ Padley et al. found that age ≤30 days and low weight (≤2500 grams) at the time of operation were associated with an OR of 10.1 (95%CI 4.7–21.5) and 8.8 (95%CI 3.6–21.2) for mortality.^20^ The main limitations in our study refer to the fact that the collection was retrospective and to the sample size.

In conclusion, knowledge about the factors that interfere with prognosis can be crucial in planning care practice, especially considering that CHD constitute, as seen in the survival curve (Figure 1), an important cause of mortality in the first year of life. In our study, later age at admission, low weight, cyanotic and complex CHD seem to contribute to a worse evolution and prognosis. Thus, initiatives that improve prenatal care and that can help in the earlier detection of CHDs can help to improve the prognosis. More studies with this population are needed to obtain indicators related to good care practices and specific outcomes, such as death.

## Data Availability

The database that originated the article is available with the corresponding author.
